# Pyrolyzed Substrates Induce Aromatic Compound Metabolism in the Post-fire Fungus, *Pyronema domesticum*

**DOI:** 10.3389/fmicb.2021.729289

**Published:** 2021-10-27

**Authors:** Monika S. Fischer, Frances Grace Stark, Timothy D. Berry, Nayela Zeba, Thea Whitman, Matthew F. Traxler

**Affiliations:** ^1^Department of Plant and Microbial Biology, University of California, Berkeley, Berkeley, CA, United States; ^2^Department of Soil Science, University of Wisconsin-Madison, Madison, WI, United States

**Keywords:** aromatic hydorcarbons, RNAseq, fire, carbon metabolic activity, PyOM, filamenous fungi

## Abstract

Wildfires represent a fundamental and profound disturbance in many ecosystems, and their frequency and severity are increasing in many regions of the world. Fire affects soil by removing carbon in the form of CO_2_ and transforming remaining surface carbon into pyrolyzed organic matter (PyOM). Fires also generate substantial necromass at depths where the heat kills soil organisms but does not catalyze the formation of PyOM. *Pyronema* species strongly dominate soil fungal communities within weeks to months after fire. However, the carbon pool (i.e., necromass or PyOM) that fuels their rise in abundance is unknown. We used a *Pyronema domesticum* isolate from the catastrophic 2013 Rim Fire (CA, United States) to ask whether *P. domesticum* is capable of metabolizing PyOM. *Pyronema domesticum* grew readily on agar media where the sole carbon source was PyOM (specifically, pine wood PyOM produced at 750°C). Using RNAseq, we investigated the response of *P. domesticum* to PyOM and observed a comprehensive induction of genes involved in the metabolism and mineralization of aromatic compounds, typical of those found in PyOM. Lastly, we used ^13^C-labeled 750°C PyOM to demonstrate that *P. domesticum* is capable of mineralizing PyOM to CO_2_. Collectively, our results indicate a robust potential for *P. domesticum* to liberate carbon from PyOM in post-fire ecosystems and return it to the bioavailable carbon pool.

## Introduction

Wildfires can have substantial effects on nutrient cycling (Wan et al., 2001; Nave et al., 2011) and community composition both above- and belowground (Bond and Keeley, 2005; Pressler et al., 2018), making them important drivers of ecosystem processes (McLauchlan et al., 2020). Furthermore, wildfires are increasing in frequency and severity in many regions of the world (Flannigan et al., 2009). Independent of soil type, wildfires have been shown to decrease the total amount of carbon in surface soils through combustion, releasing it as carbon dioxide, while much of the remaining carbon is transformed into black carbon or pyrogenic organic matter (PyOM; Certini, 2005; Knicker et al., 2005; Singh et al., 2012; Santín et al., 2015; Araya et al., 2017). PyOM encompasses a heterogeneous spectrum of compounds, but is predominantly composed of aromatic and polyaromatic compounds, depending on the source material, the temperature, and duration of pyrolysis (Czimczik and Masiello, 2007; Keiluweit et al., 2010; Bird et al., 2015). PyOM is generally thought of as being relatively recalcitrant, with PyOM sometimes persisting for hundreds or thousands of years (Singh et al., 2012; Bird et al., 2015). During intense wildfires, soil surface temperatures generally reach >500–800°C, and the temperature declines dramatically within the top 5cm of soil (Beadle, 1940; DeBano et al., 1979; Santín et al., 2016). While organic matter in surface soils may be completely combusted or pyrolyzed during fire, in deeper soil layers, non-pyrolyzed organic carbon is released where the heat from fire was enough to kill cells, but not hot enough for combustion or to catalyze the formation of PyOM. This soil layer defined by a heat-induced release of nutrients has been termed the “necromass zone” (Knicker et al., 2005; Bruns et al., 2020). Thus, post-fire soils often contain surface layers infused with PyOM, and necromass zones with abundant organic matter directly below. Early microbial colonizers of post-fire soils may exploit either or both PyOM and necromass as a key carbon source. However, relatively little is known about how the metabolism of these respective carbon sources may drive post-fire microbial succession and community recovery.

Many microorganisms are able to metabolize polyaromatic compounds with similarities to those found in PyOM, either completely or incompletely (Ghosal et al., 2016). For example, white-rot fungi have been particularly well studied for their ability to metabolize the phenolic polymer lignin. These fungi leverage a combination of peroxidases, laccases, and monooxygenases to initiate the degradation of lignin and other polyaromatic compounds (Martínez et al., 2005; Silva et al., 2009; Cerniglia and Sutherland, 2010; Janusz et al., 2017; Ostrem Loss et al., 2019). Non-lignolytic fungi rely primarily on monooxygenases, especially cytochrome P450 monooxygenases, coupled with epoxide hydrolases to initiate the degradation of complex polyaromatic compounds (Cerniglia and Sutherland, 2010; Ghosal et al., 2016; Nagy et al., 2017; Ostrem Loss et al., 2019). Several common soil fungi have also been shown to degrade polyaromatic compounds (Cerniglia and Sutherland, 2010). These fungi include *Neurospora crassa*, which emerges from burned wood shortly after fire, and *Morchella conica*, which is a relative of pyrophilous *Morchella* species that often co-occur with *Pyronema* species (Lin and Kapoor, 1979; Gramss et al., 1999; Davis, 2000; Hughes et al., 2020).

**Figure 1:**
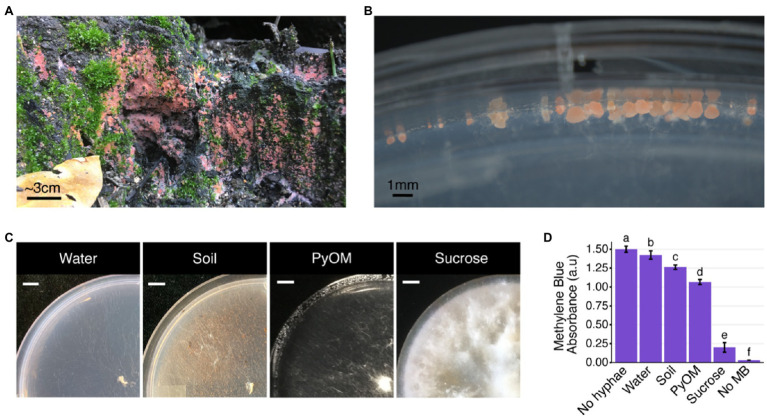
Pyronema growth on natural and laboratory substrates. **(A)** *Pyronema* sp. fruiting on burned soil 6months after the 2018 Camp Fire began near Paradise, California, United States. **(B)** *Pyronema domesticum* DOB7353 ascocarps on the edge of a water agar plate. **(C)** *Pyronema domesticum* DOB7353 growing on four different agar media treatments: water agar with no added nutrients (“Water”), wildfire-burned soil collected near the original isolation site for *P. domesticum* DOB7353 (“Soil”), 750°C White Pine wood char [“pyrolyzed organic matter (PyOM)”], or Vogel’s minimal medium with sucrose as a carbon source (“Sucrose”). All plates were inoculated in the center of the plate (bottom-right corner of photo) with mycelium from a 6-mm-diameter punch of an actively growing *P. domesticum* colony. Scale bar=1cm. **(D)** Average amount of *P. domesticum* DOB7353 biomass on one full plate as show in **(C)**, quantified by measuring the amount of Methylene Blue (MB) stain remaining after absorption by *P. domesticum* hyphae. All treatments are significantly different from each other, lowercase letters above each bar indicate significantly different groups (ANOVA+Tukey’s test, *p*<0.0001, *n*=5, error bars=SD). The “No hyphae” control is pure 0.2mMMB without any biomass treatment. The “No MB” control is a blank well.

Fruiting bodies of the genus *Pyronema* are among the first macrofungi to emerge from burned soil, doing so within weeks to months after fire (Seaver, 1909; Adamczyk et al., 2012; Bruns et al., 2020; Hughes et al., 2020; Figures 1A,B). There are two currently accepted species of *Pyronema*: *P. domesticum* and *P. omphalodes* (= *P. confluens*), both of which rapidly dominate post-fire fungal communities (Bruns et al., 2020). A recent ITS amplicon community analysis showed that *Pyronema* reads, which made up less than 1% of reads (0.91%) prior to fire achieved a post-fire average relative abundance of 60.34% (Bruns et al., 2020). Both *P. domesticum* and *P. omphalodes* were isolated from fruiting bodies that appeared within months after the catastrophic 2013 Rim Fire in the pine forest of Stanislaus National Forest, which boarders Yosemite National Park (California, United States; Bruns et al., 2020). *In vitro*, *Pyronema* has a rapid growth rate, but has historically been considered a poor competitor with other soil fungi (El-Abyad and Webster, 1968a,b). Thus, a key question is: what carbon source is used by *Pyronema* to achieve such high relative abundance post-fire? Does *Pyronema* simply exploit the available necromass, or do they have the ability to metabolize PyOM as well? Given the dominant status and their early emergence after fire, *Pyronema* likely play a critical role in the first steps of post-fire succession. Thus, the possibility that *Pyronema* might contribute to the mineralization of PyOM has far-reaching implications for carbon cycling within post-fire soil communities.

In this work, we investigated the hypothesis that early successional pyrophilous fungi such as *Pyronema* metabolize PyOM. To do so, we measured biomass, sequenced the transcriptome (RNAseq), and measured CO_2_ efflux from *P. domesticum* grown on agar media with various carbon sources, including PyOM and burned soil collected from a frequent and high-intensity wildfire site (Boisramé et al., 2017). When grown on media containing burned soil or PyOM, *P. domesticum* produced significant biomass, activated a diverse suite of cytochrome P450 and FAD-dependent monooxygenases, and comprehensively induced pathways for aromatic substrate utilization. Lastly, we confirmed that *P. domesticum* mineralized PyOM by measuring CO_2_ emissions of *P. domesticum* grown on ^13^C-labeled PyOM. Collectively, our results demonstrate the potential for *P. domesticum* to liberate carbon from PyOM, assimilate it into biomass, and mineralize it to CO_2_. Thus, pioneering organisms such as *P. domesticum* may play an important role in the short-term reintegration of PyOM into biologically available carbon in post-fire ecosystems.

## Materials and Methods

### Pyrogenic Organic Matter Production

Pyrogenic organic matter was produced from *Pinus strobus* (L.; eastern white pine) wood chips <2mm at 750°C in a modified Fischer Scientific Lindberg/Blue M Moldatherm box furnace (Thermo Fisher Scientific, Waltham, MA, United States) fitted with an Omega CN9600 SERIES Autotune Temperature Controller (Omega Engineering Inc., Norwalk, CT, United States). We modified the furnace and adapted the PyOM production design developed by Güereña et al. (2015). Briefly, the feedstock was placed in a steel cylinder inside the furnace chamber and subjected to a continuous argon gas supply at a rate of 1L min^−1^ to maintain anaerobic conditions during pyrolysis. The heating rate for production of PyOM was kept constant at 5°C min^−1^. We held the temperature constant for 30min once 750°C was reached, after which the PyOM was rapidly cooled by circulating cold water in stainless steel tubes wrapped around the steel cylinder. The PyOM was ground using a mortar and pestle and sieved to collect PyOM with particle size <45μm.

### Fungal Strain and Biomass Quantification

*Pyronema domesticum* DOB7353 (Bruns et al., 2020) agar plugs were transferred from long-term storage water stocks onto a sucrose agar plate (described below) overlaid with cellophane, and incubated at room temperature for ~5–7days. Then, a 6-mm-diameter transfer tube was used to transfer equivalent amounts of mycelium (without agar or cellophane) from the initial growth plate to inoculate each experimental agar plate. Cellophane acts as a physical barrier to ensure no agar is transferred to the experimental plates, and to facilitate easy harvesting of all biomass without agar contamination. The integrity of the cellophane is maintained throughout growth, and we never observed physical cellophane degradation after incubation with *P. domesticum*. Cellulase-encoding genes were annotated in the *P. domesticum* genome *via* the CAZy database, and we have included this CAZyme information in the Supplementary Data.

We inoculated *P. domesticum* hyphae onto four different experimental treatments: 1.5% agar media treatment plates overlaid with cellophane, Vogel’s Minimal Medium (Vogel, 1956) agar containing 20g L^−1^ sucrose (“sucrose”), 10gL^−1^ 750°C PyOM agar (“PyOM”), 10gL^−1^ wildfire-burned soil agar (“soil”), and water agar (“water”). Burned soil was collected from 0 to 10 cm in Illilouette Creek Basin (Boisramé et al., 2017) *via* an ethanol-sterilized shovel, and homogenized in plastic zip-top bags. Burned soil was *x*-ray sterilized (Steris, Petaluma, CA, United States), which used a Bremsstrahlung process to generate photons with an energy of 5–7MeV. Both PyOM and soil were added to agar media after autoclaving. Both agar media and cellophane submerged in ddH_2_O were autoclaved on a liquid cycle at 121°C and ~15–20psi for 30min.

*Pyronema domesticum* was allowed to grow for 4days until it completely covered the plate on each agar media treatment described above (sucrose, PyOM, soil, and water). All biomass from each plate was harvested by scraping with a spatula, immediately weighed, and then mixed with 500μl 0.2mM Methylene Blue (M9140, MilliporeSigma) in a 1.5ml microcentrifuge tube. We adapted Fisher & Sawers’ Methylene Blue (MB) biomass quantification protocol (Fischer and Sawers, 2013). Briefly, tubes of MB-stained biomass were heated at 80°C for 5min, vortexed at maximum speed for 10min, and then heated again at 80°C for 5min. Mycelia were pelleted by centrifugation for 10min at maximum speed in a standard microcentrifuge. About 50μl of the supernatant was combined with 200μl ddH_2_O and then absorbance was measured at 660nm. Blank wells and wells containing 0.2mMMB were included as controls.

### RNA Extraction and Sequencing

Mycelia were harvested from a total of nine replicate plates for each treatment (as described above). Mycelia from sets of three plates were pooled, resulting in three replicate samples for RNA extraction and sequencing. Pooled mycelia were immediately flash frozen with liquid nitrogen. Cells were lysed by bead-beating with 1ml TRIzol (Ellison et al., 2011). Nucleosomes were removed by gently shaking for 5min at room temperature. About 200 μl chloroform was added, briefly bead-beaten, and then centrifuged to pellet cell debris. The aqueous phase was then used for RNA purification with the Zymo Direct-zol RNA MiniPrep kit (Cat. No. R2050). The qb3 facility at University of California, Berkeley, quantified RNA quality and concentration *via* Bioanalyzer and then carried out library preparation and sequencing on an Illumina NovaSeq 6000 Platform.

### RNAseq Data Analysis

Raw reads were manually inspected for quality using FastQC v0.11.5 and then trimmed and quality filtered with Trimmomatic v0.36 (Bolger et al., 2014). HISAT2 v2.1.0 (Kim et al., 2019) mapped quality reads to the *P. domesticum* DOB7353 v1.0 genome (Nordberg et al., 2014; Bruns et al., 2020). Raw counts per gene were generated with HTSeq v0.9.1 (Anders et al., 2015). Raw counts were normalized, a principal component analysis (PCA) plot was generated, and differential expression was calculated with DESeq2 v1.24.0 on R v3.6.1 (Love et al., 2014; R Core Team, 2020). To determine whether expression profiles were significantly different across treatments, we used PERMANOVA from the adonis() function from the vegan package v2.5-7 (Oksanen et al., 2020). Functional gene annotations (including CAZymes) were downloaded from the Joint Genome Institute’s Mycocosm portal (Nordberg et al., 2014). Additional annotation of specific genes was performed *via* protein-BLAST.

### ^13^C-Labeled PyOM and Respiration Experiment

^13^C-labeled 750°C PyOM was produced from *P. strobus* as described above, except the biomass was from ^13^C-labeled seedlings. The ^13^C label was incorporated by pulse-labelling 2-year-old *P. strobus* seedlings with ^13^CO_2_, resulting in a δ^13^C value (relative to the standard vPDB) of +833.11‰ in the PyOM. We incubated the *P. domesticum* on ^13^C-labeled 750°C PyOM agar (10gL^−1^ PyOM) in 118.29ml Mason jars, fitted with gas-tight lines, and connected to an automated sample analyzer (“multiplexer”) that automatically samples the jar headspaces at regular intervals and quantifies the amount and isotopic signature of the headspace CO_2_ in a Picarro cavity ringdown spectrometer (multiplexer described in detail in Berry et al., 2021). To conserve limited labeled material while maintaining moisture in the media, we layered 10ml PyOM media over 30ml water agar in the Mason jars. *Pyronema domesticum* was inoculated using a punch from an identical ^13^C-labeled 750°C PyOM agar plate. The jars were sealed and connected to the multiplexer, where they were measured every 48–72h for 57days. Between measurements, jar headspace was flushed with a 20% O_2_, 80% N_2_, and 400ppm CO_2_ gas mix designed to represent atmospheric conditions. Measurement frequency was such that jars did not become oxygen-depleted. We used five replicates of *P. domesticum*-inoculated plates and five replicates of control uninoculated plates.

CO_2_ emissions were partitioned between sources using stable isotope partitioning and the following equation (Balesdent and Mariotti, 1996):

*f_A_* = (*δ_Total_*-*δ_B_*)/(*δ_A_*-*δ_B_*)

where *f_A_* is the fraction of total CO_2_ emissions from source A and *δ* represents the δ^13^C signature of the total (*δ_Total_*), source A (*δ_A_*), or source B (*δ_B_*). To calculate the CO_2_ that was released specifically due to the presence of *P. domesticum*, we subtracted the effects of abiotic sorption of CO_2_ by PyOM from the total CO_2_ based on the emissions from the uninoculated jars, adjusting the isotopic signature accordingly. To determine the portion of the remaining biotic emissions that were derived specifically from PyOM, we then partitioned the remaining CO_2_ between PyOM and non-PyOM sources, using the δ^13^C value of the PyOM and the δ^13^C value of media-derived CO_2_ evolved from control, *P. domesticum*-inoculated water agar plates.

### Data Availability

The datasets presented in this study can be found in online repositories. We have provided an Excel file in the supplemental materials associated with the article, which details the results of our differential expression analysis and functional category assignment. FASTQ raw RNAseq data are publicly available at SRA accession PRJNA662999. Lastly, full code used for processing gas data is available at github.com/whitmanlab.

## Results

### Pyrolyzed Substrates Induce a Distinct Transcriptional Response

To test the transcriptional response of *P. domesticum* to fire-affected substrates, we grew *P. domesticum* on two agar media treatments containing burned substrates and on two control agar media treatments. Both burned treatments shared similarities with the severely burned pine forest in the Sierra Nevada (CA, United States) from which *P. domesticum* was originally isolated. These burned substrates were 750°C *P. strobus* wood PyOM and wildfire burned soil that was collected near the original isolation site of *P. domesticum*. We observed distinct differences in the macroscopic growth pattern of *P. domesticum* when grown on the four different agar media treatments; PyOM, burned soil, sucrose, and water agar (Figure 1C). After inoculating agar treatment plates with equivalent amounts of mycelia and incubating for 4days, a substantial amount of biomass was produced on sucrose (Figures 1C,D; Supplementary Figure S1). Growth on PyOM and, to a lesser extent, burned soil both produced an intermediate amount of biomass. Notably, *P. domesticum* has a tufted or fluffy macroscopic morphology on sucrose and to a lesser extent, PyOM. Lastly, there was observable growth on water agar, but biomass production was minimal (Figures 1C,D; Supplementary Figure S1). These data illustrate that the sucrose and water treatments are functional experimental controls for robust growth and minimal growth, respectively.

**Figure 2:**
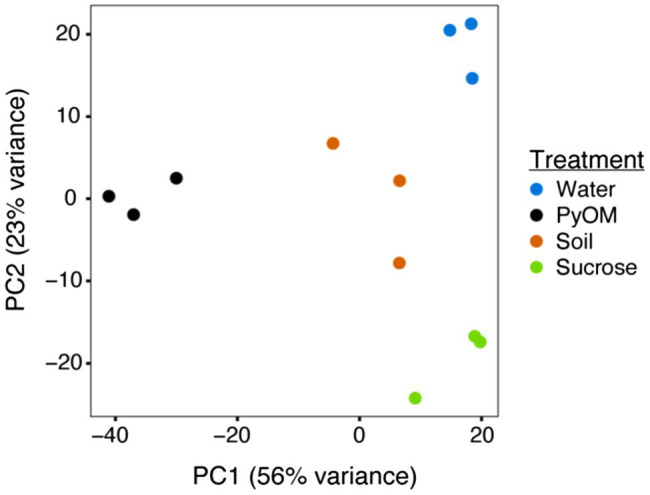
Pyrolyzed substrates induce expression of distinct sets of genes in *P. domesticum*. Principal Component Analysis (PCA) plot illustrating the variation between each sample transcriptome (normalized expression values). Prior to RNA extraction, *P. domesticum* DOB7353 was grown in triplicate on four different agar media treatments.

After 4days of growth on each substrate, the biomass from each treatment was harvested, and RNA was extracted for sequencing. PCA of these transcriptomes (Figure 2) illustrates the significant differences between treatments (PERMANOVA, *p*=0.001, *n*=3). Across PC2 (23% of variation), the transcriptomes from the water and sucrose conditions were at opposite ends, while transcriptomes from the PyOM and burned soil were located at an intermediate point near the origin. A possible explanation for this distribution is that PC2 describes the overall amount of bioavailable carbon and other nutrients. Water agar representing starvation contains the least amount of nutrients, the PyOM and soil containing intermediate amounts, and sucrose agar containing the most. Across PC1 (56% of variation), the PyOM-associated transcriptomes were located at one end of the axis, while transcriptomes from water and sucrose conditions were at the opposite end. The burned soil transcriptomes were at an intermediate position, closer to the sucrose and water conditions than PyOM. One possibility is that PC1 reflects the amount of PyOM present in the medium, since the PyOM medium contained the most, burned soil contained less, and sucrose and water media lacked any at all. Together, these results indicate that the transcriptional response of *P. domesticum* to burned or pyrolyzed substrates is significantly different from the response to water or sucrose, and the response to PyOM is particularly distinct.

### Starvation Stress Induces a Broad Transcriptional Response

**Figure 3:**
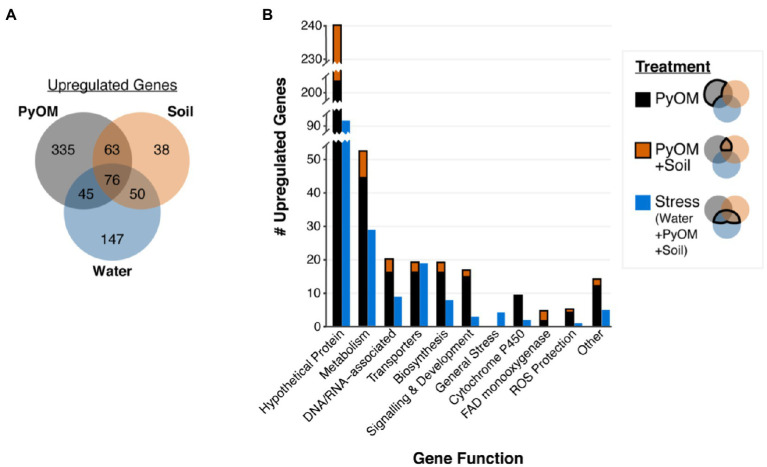
Pyrolyzed substrates induce expression of genes involved in stress response and PyOM metabolism. **(A)** Venn diagram showing the number of significantly upregulated genes in each treatment compared to sucrose (adjusted value of *p*<0.01, fold change>4, *n*=3). **(B)** Number of significantly upregulated genes compared to expression on sucrose in each functional gene category (adjusted value of *p*<0.01, fold change>4, *n*=3). Functional gene categories were determined *via* KEGG, GO, and pfam annotations. Stacked black and orange bars indicate the number of genes upregulated on PyOM alone (black) or the overlap between PyOM and soil (orange and black). We defined stress-response genes as those which are upregulated on water agar. Blue bars indicate the number of genes that are upregulated on both water and burned or pyrolyzed substrates for each functional category.

Growth on water agar triggered a broad starvation stress response in *P. domesticum* (Supplementary Data). Compared to sucrose, on water agar we observed significant upregulation of 318 genes (Figure 3A), including 31 transporters and 86 genes involved in the metabolism of diverse substrates, including the catabolism of amino acids and nucleotides (adjusted value of *p*<0.01, fold change>4, *n*=3; Supplemental Data). Several general stress response genes were also induced on water agar compared to sucrose; specifically, seven different heat shock proteins and two proteins involved in programmed cell death. Surprisingly, invertase, the enzyme that hydrolyzes sucrose, was not significantly downregulated on water compared to sucrose (adjusted value of *p*=0.14, fold change=1.8, *n*=3). In contrast to the 318 genes that were upregulated on water compared to sucrose, there were only 94 genes significantly upregulated on sucrose compared to water, including a sugar/hydrogen symporter, and 23 genes involved in primary metabolism, biosynthesis, and development (Supplementary Data). Taken together, these data demonstrate that growth on water agar induces a stress response program that includes genes involved in catabolism of macromolecules and scavenging for alternative nutrient sources. In contrast, growth on sucrose allows for a more streamlined transcriptome focused on growth powered by the metabolism of simple sugars.

### The Transcriptional Response to Pyrolyzed Substrates Is Characterized by Genes Involved in Stress Tolerance, Metabolism, and Growth

To examine the nutritional and metabolic response to burned or pyrolyzed substrates, we calculated differential expression of genes in each treatment compared to sucrose and used functional gene annotations to categorize genes that were significantly upregulated at least 4-fold (Figure 3, for downregulated genes see Supplementary Figure S2). We observed the largest shift in gene expression on 750°C PyOM with a total of 519 significantly upregulated genes (Figure 3A). Around 227 genes were upregulated on burned soil, and the majority (189 genes) of those overlapped with genes induced on PyOM and/or water (adjusted value of *p*<0.01, fold change>4, *n*=3). We note that invertase was significantly downregulated on PyOM compared to sucrose (adjusted value of *p*=1.17E-7, fold change=−9.9, *n*=3), and to a lesser extent on soil compared to sucrose (adjusted value of *p*=0.02, fold change=−5.7, *n*=3).

The 171 genes that were induced on water and at least one of the two substrates containing PyOM (burned soil and PyOM) characterized a stress response associated with decreased nutrient availability. Among these 171 genes are 19 transporters and four general stress response genes including two heat shock proteins (Figure 3B; Supplementary Data). We observed signatures of nitrogen stress in the water, PyOM, and soil conditions compared to sucrose minimal medium, which contains ammonium nitrate as a nitrogen source. These putative nitrogen stress-responsive genes include genes involved in ammonium production, nitrogen metabolism, and a putative ortholog (gene_1304) of the conserved *Aspergillus nidulans* transcription factor TamA (Supplementary Data). TamA is a conserved stress-responsive regulator of nitrogen metabolism (Small et al., 1999). Lastly, the gene_6383 encodes a putative laccase and was upregulated 26–60 fold on water, soil, and PyOM, compared to sucrose. Gene_6383 was the only laccase gene that was identified in the *P. domesticum* genome *via* pfam, KEGG, and GO analyses.

The 63 genes that were induced in common between PyOM and burned soil, excluding water, characterize a common response to pyrolyzed substrates (Figure 3). In addition, 335 genes were uniquely upregulated in response to PyOM, and the 38 genes uniquely upregulated on burned soil were almost entirely annotated as hypothetical proteins (Supplementary Data). After “hypothetical,” the next category with the most genes was that of metabolism, which we address in the subsequent section. We note that PyOM-responsive genes included nine Cytochrome P450 monooxygenases and four FAD monooxygenases. Cytochrome P450 oxidation of aromatic compounds often results in the formation of toxic epoxides and reactive oxygen species (ROS). On both substrates containing PyOM, we observed upregulation of genes involved in ROS protection (Figure 3A). However, neither of the two epoxide hydrolases annotated in the *P. domesticum* genome exhibited any significant changes across our treatments (Supplementary Data). Lastly, we observed an enrichment of genes involved in biosynthesis (e.g., synthesis of amino acids, fatty acids, and membrane lipids), development, and signaling that were upregulated specifically in the presence of PyOM. Taken together, these data indicate that, as expected, growth on PyOM is more stressful than growth on sucrose. Beyond a general stress response, the *P. domesticum* response to burned or pyrolyzed substrates includes the activation of a large set of genes, including those involved in metabolism, oxidation of aromatic substrates, and protection from ROS.

### PyOM Induces a Coherent Set of Metabolic Pathways for Aromatic Compound Degradation in *P. domesticum*

**Figure 4:**
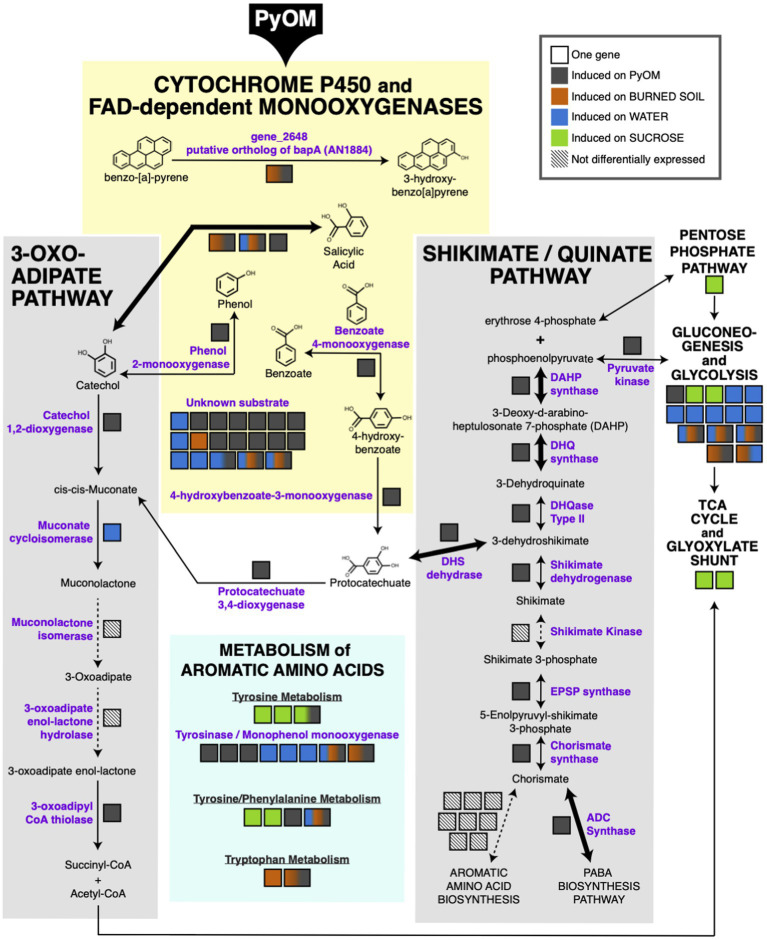
Metabolic map highlighting aromatic compound metabolism induced by growth on pyrolyzed substrates. Significantly upregulated genes mapped onto the canonical pathways for aromatic compound metabolism (adjusted value of *p*<0.01, fold change>2, *n*=3). Bolded arrows indicate a fold change>8 on PyOM compared to sucrose. Each gene is indicated as a black-outlined box, and the proteins encoded by these genes are indicated as purple text. The color fill of the box indicates the condition(s) in which the gene was upregulated. Multi-colored boxes are slightly larger than mono-color boxes to increase visibility of the colors and to highlight genes that are induced in more than one condition. Diagonal parallel lines within a box and associated dashed lines indicate genes that were expressed, but not differentially expressed under the tested conditions.

The results in the previous section indicate that PyOM may prompt a restructuring of metabolism in *P. domesticum*. In Figure 4, we mapped the significantly upregulated genes in *P. domesticum* (adjusted value of *p*<0.01, fold change>2, *n*=3) onto the canonical pathways for aromatic compound degradation and assimilation into central metabolism and other biosynthetic pathways in fungi (Tatum et al., 1954; Cain et al., 1968; Chaleff, 1974a,b; Wright, 1993; Kajander et al., 2002; Lah et al., 2011; Martins et al., 2015). All PyOM is enriched for aromatic carbon compounds because incomplete combustion of organic matter results in the formation of aromatic and polyaromatic carbon compounds (Bird et al., 2015). PyOM produced at temperatures greater than ~400°C generally has a carbon composition that is >90% aromatic, and as temperature increases, the size of polyaromatic compounds also increases (Bird et al., 2015; Ippolito et al., 2015). Here, we propose that the large cohort of cytochrome P450 and FAD monooxygenases that were induced on PyOM-containing media (compared to growth on sucrose) are the primary method that *P. domesticum* uses to initiate the degradation of polyaromatic and aromatic carbon compounds. FAD monooxygenases oxidize compounds with a single aromatic ring, whereas cytochrome P450 monooxygenases can oxidize complex polyaromatic compounds (Heine et al., 2018; Ostrem Loss et al., 2019).

One cytochrome P450 gene (gene_2648) that was upregulated on both PyOM and burned soil was identified *via* protein-BLAST as a putative ortholog of the *bapA* gene in *A. nidulans*, which was recently shown to oxidize the polyaromatic hydrocarbon benzo-[a]-pyrene (score=315, E-value=3.61e-22, 38% identity, 30.8% coverage; Ostrem Loss et al., 2019). Notably, the conserved C-terminus of the BapA protein is largely conserved in *P. domesticum*, including conserved signature motifs (highlighted in Supplemental Figure S3; Ostrem Loss et al., 2019). An additional five upregulated FAD monooxygenase genes and one cytochrome P450 monooxygenase gene were identified during genome annotation to have homology with proteins that have specific predicted substrates (salicylic acid, phenol, and benzoate; Nordberg et al., 2014). Lastly, 15 cytochrome P450 monooxygenase genes were induced at least 2-fold on PyOM-containing media that have homology to monooxygenase proteins with currently unknown substrates (Figure 4; Supplementary Table). Nearly half of these genes were strongly induced on PyOM; gene_10112, encoding a cytochrome P450 was strongly upregulated on both PyOM compared to sucrose (fold change=1910.9) and on PyOM compared to water (fold change=891.4), and six other cytochrome P450 genes were also upregulated at least 8-fold on PyOM compared to sucrose.

Assimilation of aromatic carbon into central metabolism occurs primarily *via* two pathways in fungi: either *via* protocatechuate and the shikimate/quinate pathway (Chaleff, 1974a,b), or *via* catechol and the 3-oxoadipate (=beta-ketoadipate) pathway (Wright, 1993; Martins et al., 2015). Six of seven core genes in the shikimate/quinate pathway were upregulated on PyOM compared to sucrose. In contrast, two of the five genes in the 3-oxoadipate pathway were upregulated on PyOM compared to sucrose. Notably, we observed strong upregulation on PyOM compared to sucrose of the four genes necessary to connect aromatic protocatechuate to central metabolism. These four genes encode DHS dehydrase (fold change=36.8), DHQase (fold change=4.6), DHQ synthase (fold change=955.4), and DAHP synthase (fold change=8.0). These three genes were similarly strongly upregulated on PyOM compared to water (Supplementary Data). In contrast, the genes that encode the proteins necessary for the 3-oxoadipate pathway were relatively modestly upregulated on PyOM compared to sucrose (fold change=2.8, adjusted value of *p*<0.01, *n*=3).

We also observed that genes required for the breakdown and metabolism of the three aromatic amino acids was induced differentially across all tested conditions. It is notable that upregulation of monophenol monooxygenase genes (i.e., tyrosinases) was also enriched on burned or pyrolyzed substrates and water compared to sucrose. Upregulation of central metabolism genes was generally enriched on sucrose; however, some genes involved in glycolysis and gluconeogenesis were also upregulated on water, PyOM, and soil.

In summary, when *P. domesticum* was grown on PyOM, we observed upregulation of an extensive set of monooxygenases that may initiate degradation of the aromatic components of PyOM. We also observed comprehensive induction of the shikimate/quinate and 3-oxoadipate pathways, though the shikimate/quinate pathway was much more strongly induced. These data indicate that the aromatic intermediates liberated by monooxygenases may be funneled into central metabolism and mineralized *via* the shikimate/quinate and 3-oxoadipate pathways in *P. domesticum*.

### *Pyronema domesticum* Mineralizes PyOM-Derived Carbon to CO_2_

**Figure 5:**
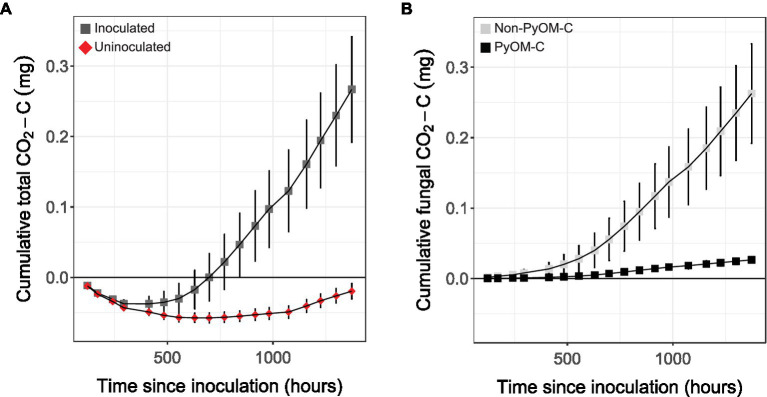
Cumulative mean CO_2_ emissions from *P. domesticum* growing on ^13^C-labeled 750°C PyOM. **(A)** Mean cumulative CO_2_ measured over time from the enclosed headspace of jars containing either sterile (uninoculated, red diamonds) 750°C PyOM agar, or identical plates inoculated with *P. domesticum* (inoculated, dark grey squares; *n*=5, error bars=SE). **(B)** Mean cumulative CO_2_ from *P. domesticum* inoculated jars, normalized by the uninoculated controls, and then partitioned into PyOM-derived C (black squares) and non-PyOM-derived C (light grey squares) using ^13^C partitioning (*n*=5, error bars=SE).

To conclusively determine whether *P. domesticum* was able to mineralize PyOM carbon, we cultivated it on agar plates supplemented with ^13^C-labeled 750°C PyOM and quantified CO_2_ emissions (Figure 5). In uninoculated control jars, we observed a net sorption of CO_2_ by the PyOM agar medium. In the headspace of gas-tight jars inoculated with *P. domesticum* (*n*=5), we observed a cumulative increase in the production of both total CO_2_ (Figure 5A) and ^13^C-labeled PyOM-derived CO_2_ (Figure 5B) over 10weeks of observation. This result indicates that *P. domesticum* mineralized some of the PyOM by converting the carbon from 750°C PyOM into CO_2_. We also observed an accumulation of non-^13^C-labeled CO_2_ in the inoculated jars, indicating that *P. domesticum* also mineralized carbon from non-PyOM sources. The CO_2_ originating from non-PyOM sources in our experiments exceeded the amount from PyOM by ~4.5 fold. Nevertheless, taken together, these results indicate that *P. domesticum* is capable of mineralizing carbon from both PyOM and non-PyOM sources under the experimental conditions used here. Moreover, CO_2_ production by *P. domesticum* more than offset the effect of abiotic CO_2_ sorption by PyOM media.

## Discussion

Fungi in the genus *Pyronema* are pioneer species that rapidly dominate fungal communities in post-fire soils (Bruns et al., 2020). Thus, *Pyronema* have the potential to directly influence the trajectory of post-fire community succession and associated nutrient cycling dynamics. Here, we investigated the transcriptional response of *P. domesticum* on four different agar treatments: 750°C PyOM, wildfire burned soil, sucrose minimal medium, and water. Our results indicate that burned or pyrolyzed substrates induce transcription of a comprehensive set of genes that together function to metabolize aromatic and polyaromatic compounds found in PyOM. Additionally, we demonstrated the mineralization of PyOM into CO_2_ by *P. domesticum*, consistent with the notion that this organism is capable of directly metabolizing high-temperature PyOM.

*Pyronema* are barely detectable in soil before fire, become prevalent soon after fire, and then rapidly decline within weeks (Seaver, 1909; Bruns et al., 2020). The form taken by *Pyronema* between fire events is largely obscure. *Pyronema* may simply exist as dormant ascospores or sclerotia that require the heat and/or chemical changes associated with fire to break dormancy and initiate germination (Emerson, 1947; El-Abyad and Webster, 1968a). For example, plant-derived polysaccharides such as hemicellulose burned at high temperatures result in the formation of furfural, which triggers germination of *N. crassa* ascospores (Eilers and Sussman, 1970; Feldman et al., 2019). One recent hypothesis suggests that pyrophilous fungi may live as endophytes for the majority of their life history, abandoning their plant hosts after they are killed by fire (Raudabaugh et al., 2020). Regardless of how *Pyronema* live pre-fire, post-fire *Pyronema* are clearly poised to take full advantage of an open niche. Past work has shown that *Pyronema* are poor competitors, and they are also capable of growing rapidly on a diversity of substrates (i.e., burned soil, steam-treated soil, several different soil types, heat-treated plaster, and agar media containing various nutrients; El-Abyad and Webster, 1968a,b). These data point toward the notion that *Pyronema* are generalists.

During intense forest fires, the organic material in the topmost layer of soil is heavily pyrolyzed, ultimately containing a significant amount of PyOM, with patches where the soil surface is covered in a layer of pure PyOM (Santín et al., 2015). PyOM is a heterogeneous material composed of complex aromatic and polyaromatic carbon compounds (Wan et al., 2001; Nave et al., 2011; Bird et al., 2015). Beyond PyOM, this top layer of soil is depleted of easily-metabolized C sources and other macronutrients (Araya et al., 2017). A secondary layer of soil beneath the top layer is heated to a point that causes widespread death of the resident microbial/invertebrate soil fauna, leading to a layer rich in necromass (composed of simple bioavailable carbon and other nutrients) that is not pyrolyzed (Knicker et al., 2005; Bruns et al., 2020). Carbon found in either layer could be targeted by *Pyronema*.

The notion of *Pyronema* as generalists might suggest that they would be most likely to exploit the readily available carbon in the necromass layer. In contrast, growth on PyOM-containing agar, the metabolic restructuring at the transcriptional level, and production of ^13^C-labeled CO_2_ from labeled 750°C PyOM that we observed in this study indicate that *P. domesticum* is capable of metabolizing PyOM. Specifically, this metabolic restructuring includes the activation of an array of cytochromes P450 and FAD monooxygenases which may target aromatic substrates for oxidation. Additionally, we observed robust activation of the shikimate/quinate and 3-oxoadipate pathways for assimilating the resulting substrates into central metabolic pathways (Chaleff, 1974a,b; Martins et al., 2015). Epoxide hydrolase activity is often coupled with cytochrome P450 activity in polyaromatic degradation pathways (Cerniglia and Sutherland, 2010; Ghosal et al., 2016; Nagy et al., 2017; Ostrem Loss et al., 2019). Notably, we did not observe any significant differential expression of epoxide hydrolase genes in *P. domesticum*, indicating that pathway may not be relevant in *P. domesticum*. Together our results indicate that *Pyronema* may in fact be well adapted as broad generalists able to capitalize on both necromass and abundant PyOM in post-fire soils, including high-temperature PyOM, which is often assumed to be recalcitrant and lacking in bioavailable carbon (Bird et al., 2015). Furthermore, this broad generalist behavior may explain how *Pyronema* are able to rapidly dominate post-fire soil communities.

Although their prevalence is relatively short-lived, *Pyronema* grow rapidly post-fire, producing abundant biomass in the form of ascocarps and mycelia (Seaver, 1909; Adamczyk et al., 2012; Bruns et al., 2020; Hughes et al., 2020). However, both *Pyronema* ascocarps and their DNA decline rapidly after they peak in abundance following fire (Bruns et al., 2020). Some decline of *Pyronema* DNA could be explained by the starvation response that we observed on water agar (Figure 3), in which *P. domesticum* may fuel outward expansion by recycling macromolecular building blocks such as nucleotides and amino acids into a diffuse biomass aimed at exploration of environments with sparse nutrients (Nitsche et al., 2012; Ellström et al., 2015). This turn-over of biomass may account for some of the non-PyOM-derived CO_2_ detected in our experiments (Figure 5B). Alternatively, *P. domesticum* may have simply mineralized other carbon sources that were present in the agar medium, such as impurities in the agar itself. To our knowledge, terrestrial fungi lack agarases that degrade agarose, but the genomes of fungi such as *P. domesticum* do contain a suite of pectinases, some of which may target agaropectin (Balabanova et al., 2018). The data in Figure 5B demonstrate that *P. domesticum* is capable of mineralizing carbon from PyOM produced at 750°C, which yields material that is completely pyrolyzed, and hence challenging to break down biologically. Furthermore, the finding that *P. domesticum* produced CO_2_ from scarce, non-PyOM sources in the presence of abundant PyOM implies that they likely prefer to metabolize other carbon substrates over PyOM if they are available. While such a metabolic hierarchy makes sense from an energetic standpoint, it also reflects the ecology of these organisms. For example, *Pyronema* may grow abundantly on C available in the necromass layer and only switch to metabolizing PyOM after those resources are exhausted or during surface growth during fruiting body formation.

The short-lived dominance of *Pyronema* might also be explained by competition, as it appears to be a weak competitor in isolation (El-Abyad and Webster, 1968b). However, even if *Pyronema* are outcompeted and simply senesce, their DNA could linger in post-fire soil and continue to be detected *via* sequencing methods (Levy-Booth et al., 2007; Pietramellara et al., 2009; Carini et al., 2017). Thus, another possible explanation for the rapid decline of *Pyronema* DNA in post-fire soils is that *Pyronema* biomass, either living or recently senesced, is consumed by other organisms (Levy-Booth et al., 2007; Pietramellara et al., 2009). Thus, abundant *Pyronema* biomass may provide a critical nutrient source for secondary colonizers of post-fire soils, thereby laying the foundation for succession within post-fire communities. Importantly, the ability of *P. domesticum* to convert some PyOM into biomass could directly facilitate the growth of organisms that lack the ability to metabolize PyOM. Thus, *Pyronema* may provide an important mechanism for rapidly assimilating some portion of newly formed PyOM back into more readily bioavailable forms of carbon in post-fire environments. Additionally, it is possible that *Pyronema* function to favorably transform the post-fire soil environment in other ways, such as affecting pH or accessibility of other nutrients. Nevertheless, the mineralization of PyOM by the dominant early-successional fungus *P. domesticum* is likely to have broad impacts on post-fire succession and recovery in soil microbial communities.

## Data Availability Statement

The datasets presented in this study can be found in online repositories. The names of the repository/repositories and accession number(s) can be found in the article/Supplementary Material.

## Author Contributions

MF and MT conceptualized and designed this study. MF, FS, TB, and NZ conducted experiments (including optimization/validation) and collected data. MF, FS, TB, NZ, and TW processed and analyzed the data. MF, NZ, and TW generated the figures. MF wrote the first draft of the manuscript. FS, TB, NZ, TW, and MT edited and provided feedback on the original draft. MT and TW acquired essential funding and resources. MF, MT, and TW provided supervision and project management. All authors contributed to the article and approved the submitted version.

## Funding

This research was supported by the DOE Office of Science and Office of Biological and Environmental Research (BER); grant no. DE-SC0020351 to TW and MT.

## Conflict of Interest

The authors declare that the research was conducted in the absence of any commercial or financial relationships that could be construed as a potential conflict of interest.

## Publisher’s Note

All claims expressed in this article are solely those of the authors and do not necessarily represent those of their affiliated organizations, or those of the publisher, the editors and the reviewers. Any product that may be evaluated in this article, or claim that may be made by its manufacturer, is not guaranteed or endorsed by the publisher.
