# Supplementary material for: Pyrolyzed Substrates Induce Aromatic Compound Metabolism in the Post-fire Fungus, Pyronema domesticum
Source: Front Microbiol. 2021 Oct 27;12:729289. doi: 10.3389/fmicb.2021.729289 (PMC8579045; doi:10.3389/fmicb.2021.729289)
Supplement: Supplementary file 2 [file presentation_1.pdf]

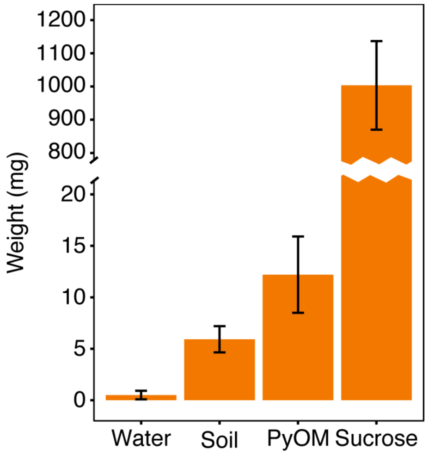

Supplemental Figure 1. *P. domesticum* biomass wet weight on agar media treatments.

Prior to Methylene Blue staining, *P. domesticum* biomass was harvested and immediately weighed. Error bars indicate standard deviation (n = 5).

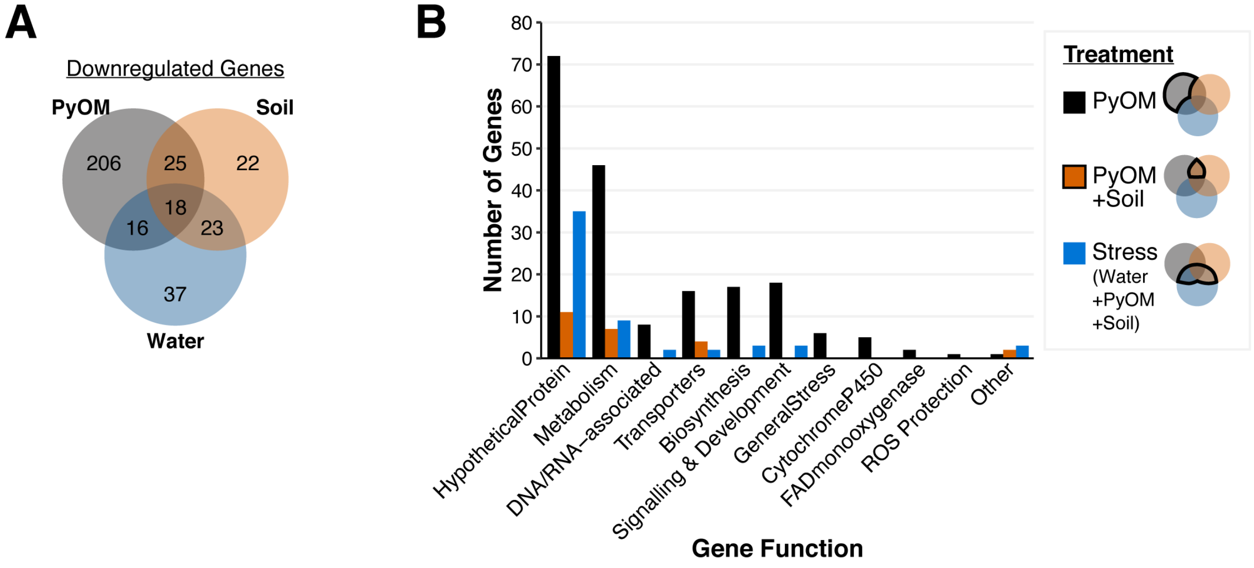

Supplemental Figure 2. Down-regulated genes on pyrolyzed substrates in *P. domesticum*.

(A) Venn diagram showing the number of significantly downregulated genes in each treatment compared to sucrose (adjusted p-value < 0.01, fold change < -4, n = 3). **(B)** Number of significantly downregulated genes compared to expression on sucrose in each functional gene category (adjusted p-value < 0.01, fold change < -4, n = 3). Functional gene categories were determined via KEGG, GO, and pfam annotations. Black bars indicate the number of genes downregulated on PyOM alone (total = 206). Orange bars indicate the number of genes downregulated in both PyOM and soil (total = 25). Blue bars indicate the number of genes downregulated on water that also overlap with soil and/or PyOM (total = 57).

```
CLUSTAL O(1.2.4) multiple sequence alignment

jgi|Pyrdom1|124492|gene_2648          MDPATIIASVKAMTPIQLGA--VGLIG----------FYILASLFTSIQTSIKSRSL---        45
AN1884                                ------------MAPIPWGMLYVSVVGYSVLTSNLFTFFVLATALTALKIVYASFLYPEY        48
                                                  *:**  *   *.::*          *::**: :*:::    *

jgi|Pyrdom1|124492|gene_2648          -----GC-QSPPWAWDPLGLRRVYNMM---RYALNHQLPLYSVKLFKDFNTKTVPIIDFG        96
AN1884                                LTPIKHIPTPPKRSWVRGN-TDTYVVISPFEGMLNWTKSVPNNGLLRYYIIGNI------        101
                                                *  :*   .   .* ::   .  **    : .  *:: :   .:

jgi|Pyrdom1|124492|gene_2648          KPGYLTCDPRNIQAALATNFKDWGF-GSARYPMLPIL-GDGIFTQDGEAWAHSRSMIRPS        154
AN1884                                -EQVLVTTPKALSELLVQNAYDYQKPESIRISLARIAGEHGILLVEGQEHKRHRKNLMPA        160
                                          *.  *: :.  *. *  *:    * *  :  *   .**:  :*:   : *. : *:

jgi|Pyrdom1|124492|gene_2648          FTKSQIADFE------------SLEEHMQEFFTTLEMTTKSDGAVSLKPLFSDLTMDFAS        202
AN1884                                FSYRHIKDLYPTFWAKSVEMVKCIEKDLQDRR------DTGDITVTVRPWASRATLDIIG        214
                                      *:  :* *:             .:*:.:*:         ..* :*:::*  *  *:*: .

jgi|Pyrdom1|124492|gene_2648          EF----LFGETA---NSLKQRREGIAETGMAHWFD--------AGMHHVTMSFNMGRLHN        247
AN1884                                LAGMDRDFGSLADPQNELAAQYHRILE-DPPLWLKLLFAAAFVLGNEELVMALPVK----        269
                                             **. *   *.*  : . * * .   *:.         * ..:.*:: :

jgi|Pyrdom1|124492|gene_2648          FWRPKEYRQSIKFVREFVDGFVHRALEERLAIGDIKEKERAKMNGGRYVFLNALTDNITD        307
AN1884                                --RNRDIAEGAKYVRQVAQQLISEK---RERIKHNPE-KAEGGGGGKDILSVALNSGNFT        323
                                        * ::  :. *:**:..: :: .    *  * .  * :    .**: ::  **...

jgi|Pyrdom1|124492|gene_2648          PIVLRNQVLNIMLAGRDTTAALLSWVLWNLARRPEVLERLKQEVAETIGVAENA--TLPT        365
AN1884                                DIELIDQMMTFLAAGHETTSSALQWSVYALCKHPDIQTRLRQEIRSNLPSVSSNDPKPIT        383
                                       * * :*::.:: **::**:: *.* :: *.::*::  **:**: ..:  ...   .  *

jgi|Pyrdom1|124492|gene_2648          WQVLKDMRYLQAVIHETLRLFPSVPFSNRIATRDTVLPYGGGSDGNAPLFAPKDTQLIYS        425
AN1884                                AEAVDSLPYLHAFCNEVLRFHPSVPITFRTTTRDTTLA---------GTLLPKGTQLTIS        434
                                       :.:..: **:*. :*.**:.****:: * :****.*            : **.***  *

jgi|Pyrdom1|124492|gene_2648          IYAMQRRPEIWGADAAEFSPDRWLRADAGKM-LREVGWGNMPFSGGPRICPGQQFALTET        484
AN1884                                PEVINHDPDLWGPDAHIFNPDRWLGPGRANTGGASSNYALMTFLHGPRSCIGQGFAKAEL        494
                                        .::: *::** **  *.*****  . .:    . .:. * *  *** * ** ** :*

jgi|Pyrdom1|124492|gene_2648          SYVVSRLLQRYQWVEKAPGESDVPSFEASLVTPPADMVEVMFTKY*--       529
AN1884                                ACLVAAMVGRFEMELADPGKKLAVRRTAT-IS-PVDGVVARLTPLDGW       540
                                      : :*: :: *::     **:. .    *: :: *.* * . :*
```

Supplemental Figure 3. Protein sequence alignment of the *A. nidulans* bapA gene (AN1884) and *P. domesticum* gene_2648. Alignment performed using the EMBL-EBI Clustal Omega web tool (Madeira et al., 2019). Yellow highlights indicate broadly conserved motifs (Ostrem Loss et al., 2019).
